# Robust contribution of decadal anomalies to the frequency of central-Pacific El Niño

**DOI:** 10.1038/srep38540

**Published:** 2016-12-05

**Authors:** Arnold Sullivan, Jing-Jia Luo, Anthony C. Hirst, Daohua Bi, Wenju Cai, Jinhai He

**Affiliations:** 1 CSIRO Marine and Atmospheric Research, Melbourne, Australia; 2 Bureau of Meteorology, Melbourne, Australia; 3 Nanjing University of Information Science and Technology, Nanjing, China

## Abstract

During year-to-year El Niño events in recent decades, major sea surface warming has occurred frequently in the central Pacific. This is distinct from the eastern Pacific warming pattern during canonical El Niño events. Accordingly, the central-Pacific El Niño exerts distinct impacts on ecosystems, climate and hurricanes worldwide. The increased frequency of the new type of El Niño presents a challenge not only for the understanding of El Niño dynamics and its change but also for the prediction of El Niño and its global impacts at present and future climate. Previous studies have proposed different indices to represent the two types of El Niño for better understanding, prediction and impact assessment. Here, we find that all popularly used indices for the central-Pacific El Niño show a dominant spectral peak at a decadal period with comparatively weak variance at interannual timescales. Our results suggest that decadal anomalies have an important contribution to the occurrence of the central-Pacific El Niño over past decades. Removing the decadal component leads to a significant reduction in the frequency of the central-Pacific El Niño in observations and in Coupled Model Intercomparison Project Phase 5 simulations of preindustrial, historical and future climate.

El Niño, the most important driver for year-to-year global climate variations, is classically referred to as substantial sea surface temperature (SST) warm anomalies in the equatorial central-eastern Pacific (EP, see https://climate.ncsu.edu/climate/patterns/ENSO.html). However, recent observations show that major warm SST anomalies of El Niño events in recent decades have frequently been confined to the central Pacific (CP) with less warm or even slight cold anomalies in the east^1^,^2^,^3^. This distinct phenomenon has been referred to as Date-line^4^, Modoki^1^, warm pool^5^ or CP El Niño^3^. It has been found that the CP and EP El Niño events have distinct weather and climate impacts around the globe^6^,^7^,^8^,^9^,^10^,^11^. The two types of El Niño are controlled by thermocline feedback and zonal surface ocean advection with different relative importance^5^,^12^. While the EP El Niño usually acts to discharge heat content from the equatorial Pacific and displays a major periodicity of around three-five years, the CP El Niño plays a weak role in modulating the equatorial upper-ocean heat content and displays a shorter periodicity of around two-four years^3^,^5^,^12^. Because of these distinct differences, various indices have been proposed in an attempt to separate the two different flavours of El Niño^1^,^3^,^4^,^5^,^13^,^14^,^15^,^16^,^17^, despite continuing debate regarding whether the two types of El Niño represent two different physical modes or just reflect a diverse continuum of El Niño or different phases of El Niño evolution^17^,^18^,^19^,^20^,^21^.

A direct way to represent the two types of El Niño is to use the conventional SST indices in the Niño4 and Niño3 areas (Methods) that approximately match the locations of the maximum SST warm anomalies of the CP and EP El Niño, respectively. However, the Niño4 and Niño3 indices themselves are highly correlated (Supplementary Table 1) and thus cannot be *naturally* separated; one has to additionally compare the magnitudes of the two indices to classify the El Niño^2^,^5^. Other popular methods are aimed at building two statistically independent indices by means of the intrinsic orthogonality of Empirical Orthogonal Functions (EOF)^1^ or rotated EOF^14^ or by transforming the Niño4 and Niño3 indices^15^ or two leading EOF principal components^13^ or by separately removing the EP and CP El Niño related SST anomalies before performing the EOF analysis^3^,^16^. The simplest of these existing methods is to transform the Niño4 and Niño3 indices but with conditional constraints^15^. That is, EP_tN3N4_ = N3 − α N4; CP_tN3N4_ = N4 − α N3, where N3 and N4 denote the Niño3 and Niño4 index respectively, and α = 0.4 if N3*N4 > 0 or α = 0 if N3*N4 ≤ 0. As a consequence, however, the EP_tN3N4_ and CP_tN3N4_ are nonlinearly related to the N3 and N4 indices.

The two indices can be further simplified by normalizing N3 and N4 and fixing α = 0.5. Namely, EP_new_ = N3_normalized_ − 0.5*N4_normalized_ and CP_new_ = N4_normalized_ − 0.5*N3_normalized_. The EP_new_ and CP_new_ index has a high correlation (about 0.9) with the EP_tN3N4_ and CP_tN3N4_ index, respectively. Their correlations with the other indices are also high (Supplementary Table 1b and c, and Supplementary Fig. 1). An advantage of the EP_new_ and CP_new_ indices over the others is that, by fixing α = 0.5, the EP_new_ and CP_new_ index has the same high correlation (0.88) with the N3 and N4 index, respectively (Supplementary Table 1a). Besides, similar to the other indices, the EP_new_ and CP_new_ indices are nearly independent from each other with a weak correlation of −0.11 (Supplementary Table 1a). They capture the different spatial patterns of the CP and EP El Niño (Supplementary Fig. 2). Additionally, the sum of the EP_new_ and CP_new_ indices represents a mixed type of El Niño and is approximately equal to the Niño3.4 index (Supplementary Fig. 2 and Supplementary Table 1a). The EP_new_ index shows a positive skewness, reflecting the fact that the EP El Niño signal is stronger than that of La Niña in the eastern Pacific. In contrast, the CP_new_ index displays a weak negative skewness, because in the central Pacific the La Niña anomalies are stronger than the El Niño anomalies^22^.

## Results

**Figure 1:**
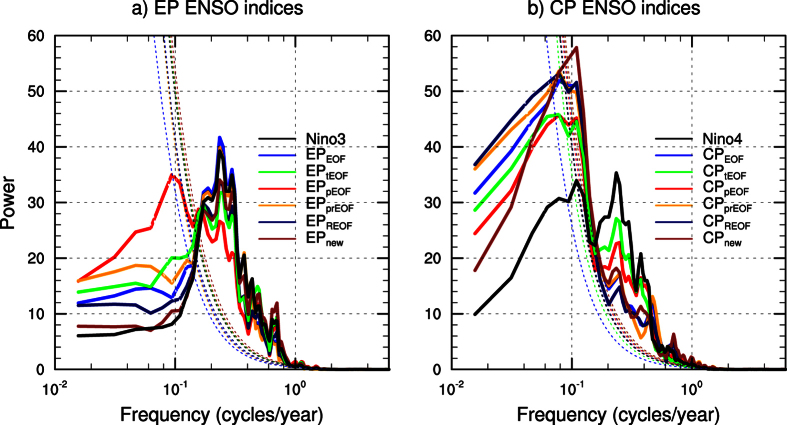
Power spectra of normalized El Niño indices during 1950–2013. Results are calculated based on (**a**) the detrended five-month running mean Niño3 and EP El Niño indices, and (**b**) the Niño4 and CP El Niño indices. The EP and CP El Niño indices (coloured lines) are computed following previous studies. Namely, the EP_EOF_ and CP_EOF_ indices are computed based on the conventional EOF method^1^. Note that the CP_EOF_ index is very similar to the El Niño Modoki index^1^. The EP_tEOF_ and CP_tEOF_ indices are generated by further transforming the principal components of the two leading EOFs^13^. The EP_pEOF_ and CP_pEOF_ indices are also computed with the EOF method but with the EP and CP El Niño-related components being separately removed *a priori* (i.e., a partial EOF approach^3^). Similarly, the EP_prEOF_ and CP_prEOF_ indices are computed with the SST anomalies partially regressed onto the Modoki and Niño3 index being separately removed before performing the EOF analysis^16^. The EP_REOF_ and CP_REOF_ indices are calculated with the rotated EOF method^14^. Finally, the EP_new_ and CP_new_ indices are generated by a simple linear transform of the Niño3 and Niño4 indices (see the text for details). The thin dashed lines indicate the 10% significance level. Results based on non-detrended indices are very similar. Note that since all the indices are normalized prior to the spectral analysis, the displayed magnitude of the variance of each period does not represent its actual magnitude. This figure is created using NCAR Command Language software package version 6.3.0 (https://www.ncl.ucar.edu).

The existing CP and EP El Niño indices have been widely used to explore possible differences in their features, underlying mechanisms, predictabilities and climate and societal impacts^1^,^2^,^3^,^4^,^5^,^6^,^7^,^8^,^9^,^10^,^11^,^13^,^14^,^15^,^16^. Here, we use spectral analysis to show that these CP and EP (including the CP_new_ and EP_new_) El Niño indices actually represent two phenomena at distinct timescales, rather than at the same interannual timescale (Fig. 1). The results indicate that most of the EP indices (except the index calculated with a partial EOF^3^) correctly capture the major spectral peak around three-to-five years (Fig. 1a). And consistent with the Niño3 index, the decadal variance in most of the EP indices is only about a half of the interannual variance (Supplementary Table 2a). In stark contrast, all the CP indices display a major peak at a decadal timescale (around 10 years); the variance captured at the interannual timescale is quite small (Fig. 1b). The decadal variance in all the CP indices is 1.6–2 times of the interannual variance (Supplementary Table 2b). This is at odds with the Niño4 index that shows comparable peak variances at periods of about four and 10 years. The result suggests that in the central Pacific, decadal signal is as important as interannual variability, consistent with previous studies^23^,^24^ that have shown decadal signal (with a similar period) is stronger in the central Pacific than that in the east. While the decadal variance of some CP El Niño indices (e.g., the Modoki index^1^) has been noticed in previous studies^1^,^12^, exact impact of decadal anomalies on the CP El Niño has not yet been assessed. Here, we aim to provide a quantitative analysis of the contribution of decadal anomalies to the frequency of the CP El Niño.

**Figure 2:**
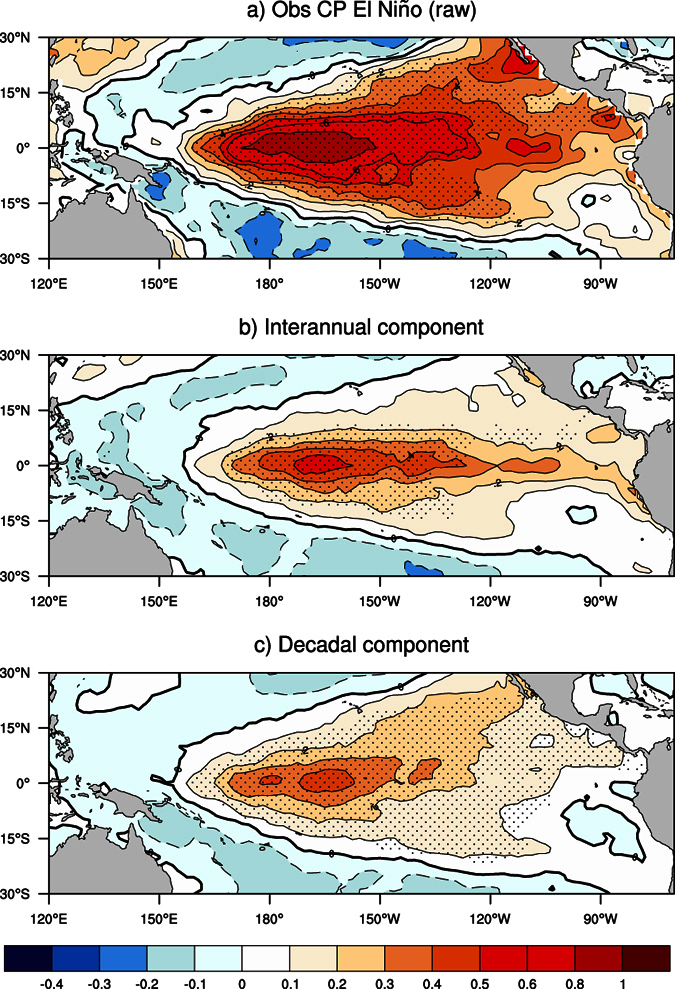
Composite maps of CP El Niños at different timescales. (**a**) Averaged SST anomalies (°C) of the CP El Niño events during 1950–2013 based on detrended HadISST observations. **(b**) and (**c**), As in (**a**), but based on the band-pass (five months to seven years) and low-pass (greater than seven years) filtered SST anomalies of the identified CP El Niño events. Stippling indicates the 5% significance level according to a Student’s t-test. Results based on non-detrended data are similar. This figure is created using NCAR Command Language software package version 6.3.0 (https://www.ncl.ucar.edu).

Because of the weak ability of all the CP indices to capture interannual El Niño, we classify the CP and EP El Niño by comparing the relative magnitudes of the Niño3 and Niño4 indices (Methods). While this method may misrepresent the influence of a mixed type of El Niño, it appears to be effective and has been widely adopted to identify the two types of El Niño^2^,^5^. We first identify CP El Niño events based on five-month running mean SST anomalies during 1950–2013, and then split the selected CP El Niño events into interannual (five months to seven years) and decadal (greater than seven years) components. The results show that, after removing the decadal component, the average strength of the selected CP El Niño events decreases substantially; only in a small area of the central Pacific do the SST anomalies still reach above 0.5 °C (the threshold of El Niño, Fig. 2b). Seven of the selected 16 CP El Niño events during 1950–2013 do not meet the 0.5 °C threshold if the decadal contributions are removed. In addition, we find that the decadal component varies over time. For instance, in the 1994/95 and 2002/03 CP El Niño cases, the decadal SST warming in the central Pacific plays a major role (Supplementary Fig. 3). In particular, the “horse-shoe” pattern of the decadal SST anomalies is reminiscent of the decadal El Niño-like variation or meridional mode^23^,^24^. Whereas, in the 2006/07 and 2009/10 CP El Niño cases, the decadal cooling in the eastern Pacific acts to dampen the interannual SST warm anomalies there, consistent with the La Niña-like climate shift in the last decades^25^,^26^. Decadal anomalies also contribute to the EP El Niño SST warm anomalies in the eastern Pacific (Supplementary Fig. 4), but their impacts are weak. Without decadal contributions, composite SST anomalies remain similar and most of the selected EP El Niño events still exceed the threshold of 0.5 °C (Supplementary Figs 4 and 7c).

Previous studies have suggested that climate change in response to anthropogenic forcing might affect the occurrence and characteristics of El Niño events^27^,^28^. In particular, studies have shown that the frequency of the CP El Niño is projected to increase under a future warmer climate^2^ but with large uncertainties^27^. We further examine the impacts of decadal and longer timescale anomalies on the CP and EP El Niño based on a variety of centennial simulations from the Coupled Model Intercomparison Project phase 5 (CMIP5) (Methods and Supplementary Table 3). Many current state-of-the-art models show a severe deficiency in reproducing a decadal peak of power spectrum in the central Pacific (Supplementary Fig. 5). Nevertheless, the ratios of decadal variance to total variance of both the Niño4 and Niño3 indices are well reproduced, and all CMIP5 simulations realistically reproduce the larger contribution of decadal anomalies in the central Pacific than that in the eastern Pacific (Supplementary Table 4).

**Figure 3:**
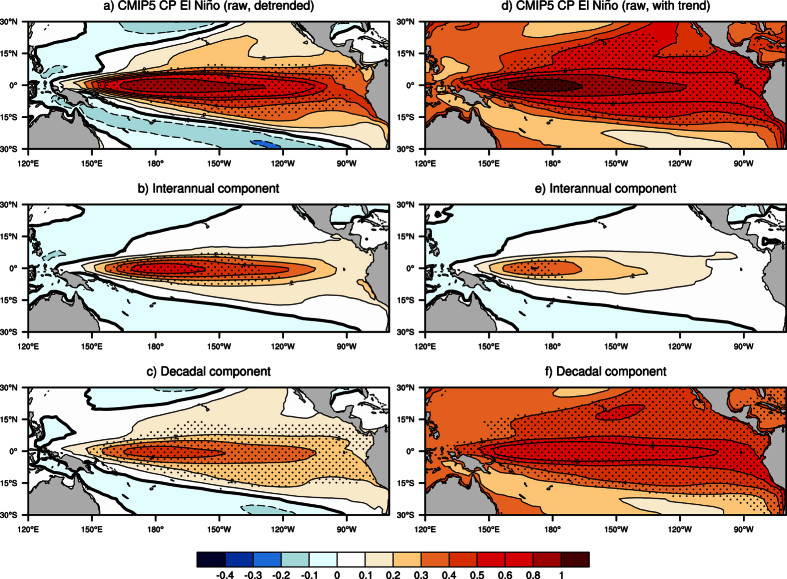
Composite maps of CP El Niños at different timescales based on CMIP5 simulations. (**a–c**) As in Fig. 2, but for detrended model outputs that include pre-industrial, historical and four RCP scenario-based simulations (Methods). In spite of the different rates of global warming, results based on each simulation are similar (Supplementary Fig. 8). (**d–f**) As in (**a–c**), but for non-detrended model outputs.

**Figure 4:**
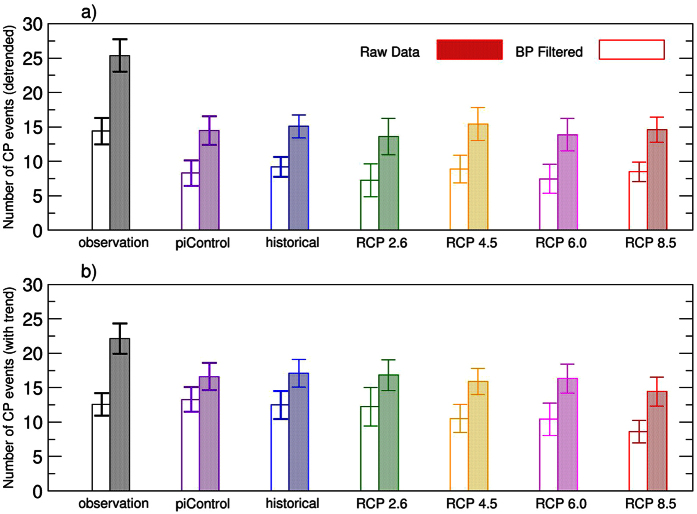
Impact of decadal anomalies on the frequency of the CP El Niño. (**a**) Frequency of the CP El Niño per 100 years based on observations and CMIP5 model simulations, determined using the detrended five-month running mean (filled bars) and band-pass (BP, five months to seven years) filtered (blank bars) SST anomalies. Error bars indicate the 95% confidence intervals. (**b**) As in (**a**), but for non-detrended SST anomalies. The frequency of the modelled CP El Niño in various simulations is underestimated compared to the observed, partly due to the models’ common deficiency in simulating the CP El Niño (i.e., the simulated El Niño signal often extends from the eastern Pacific too far to the west)^29^. This figure is created using Grace Version 5.1.23 (http://plasma-gate.weizmann.ac.il/Grace/).

Model results confirm the importance of the decadal and longer timescale signals on the CP El Niño (Fig. 3). Without the contributions of the long timescale anomalies, the average magnitude of CP El Niños is largely reduced and the frequency of the CP El Niño is also significantly decreased (Figs 3 and 4). Results are robust even if the Niño3 and Niño4 boxes are shifted westward by 10°–20° of longitude to account for the models’ common bias that shows simulated El Niño pattern extends too far to the west^29^ (Supplementary Figs 6 and 7). Adding the centennial linear trends exerted by radiative forcing does not affect results (Figs 3 and 4), except that the averaged SST anomalies of CP El Niños become positive across the entire tropical Pacific. For the EP El Niño, model results suggest that decadal and longer timescale anomalies are less important (Supplementary Figs 4 and 7), supporting observational results.

## Summary and Discussion

In summary, our results find that all of the popularly used CP El Niño indices display a major spectral peak at a decadal period rather than at interannual timescales, probably owing to the orthogonality or independence constraint that acts to mathematically minimize the correlation between the CP and EP SST anomalies. Note that many of the CP indices are able to capture weak peak variances at about two and four years; this suggests that these indices may still be useful for examining interannual variability if their decadal components are removed^12^. Further efforts are required to build a physics-based index to better represent the CP El Niño at interannual timescales. The CP El Niño is usually not as strong as the EP El Niño and is less controlled by equatorial ocean-atmosphere coupling^19^. The results presented here suggest that decadal anomalies have an important contribution to the occurrence of the CP El Niño over the past six decades^20^,^25^,^30^,^31^,^32^, and are supported by CMIP5 simulations. The EP El Niño, on the other hand, has a relatively strong intensity and is largely controlled by the oceanic thermocline feedback and the year-to-year recharge-discharge process^33^ and hence it is less impacted by slowly-varying decadal anomalies.

It is worth noting that mechanisms of the Pacific decadal/multi-decadal anomalies are complicated and remain to be poorly understood. Different hypotheses have been proposed to explain the decadal/multi-decadal anomalies in the tropical Pacific^20^,^23^,^24^,^25^,^26^,^34^,^35^,^36^,^37^,^38^,^39^,^40^,^41^,^42^,^43^. It has been argued that the El Niño-La Niña asymmetry may contribute to the decadal background change in the tropical Pacific^25^,^35^,^36^,^37^. While the strong positive skewness in the eastern Pacific implies that warm decadal anomalies there may be partly ascribed to the EP El Niño itself, the weak negative skewness in the central Pacific indicates that decadal anomalies there are less influenced by the El Niño-La Niña asymmetry. It has also been argued that multi-year persisting La Niña anomalies induced by nonlinear processes and the rectification of El Niño-Southern Oscillation (ENSO) events may contribute to the Pacific decadal/multi-decadal anomalies^38^,^39^. Previous studies have also suggested the important roles of internal ocean-atmosphere processes in generating the Pacific ENSO-like decadal/multi-decadal variations^23^,^24^,^40^,^41^. For instance, it has been found that slowly-varying ocean subsurface anomalies from the extratropics, particularly the South Pacific Ocean, may induce decadal/multi-decadal variations in the tropics^23^,^40^,^41^. Besides, recent studies has suggested that decadal/multi-decadal SST warming trends in the tropical Indian Ocean and the Atlantic, partly forced by increased greenhouse gases emissions, may also affect the Pacific climate change^26^,^34^,^42^. It has also been argued that aerosol forcing in the past decades may force ENSO-like decadal/multi-decadal anomalies in the tropical Pacific^43^.

Disentangling the complex mechanisms for the tropical Pacific decadal/multi-decadal anomalies remains to be a long-standing challenge. This is beyond the scope of this study and warrants further investigations. Improved understanding of the decadal and longer timescale anomalies, induced by either nonlinear processes and rectification of ENSO, Pacific internal mechanisms, decadal background-ENSO interactions, stochastic processes, inter-basin interactions, external radiative forcing, or the combination of these factors^20^,^23^,^24^,^25^,^26^,^30^,^34^,^35^,^36^,^37^,^38^,^39^,^40^,^41^,^42^,^43^, could improve our understanding of the CP El Niño and projections of future El Niño changes.

## Methods

### Observational and climate model data

To identify EP and CP El Niños over 1950–2013, we use the Hadley Centre Sea Ice and Sea Surface Temperature (HadISST) data set^44^. The HadISST has 1° resolution and is reconstructed based on both in situ and satellite observations. Results based on other available datasets are similar. Monthly SST anomalies are calculated relative to the monthly climatology of 1950–2013 and then smoothed with a five-month running mean to remove high-frequency noise. We also use centennial-length CMIP5 simulations^45^ (Supplementary Table 3), including: 1) pre-industrial runs from model year 101–200 (i.e., the first 100-year spin-up is discarded) with fixed preindustrial greenhouse gas forcing (piControl), 2) historical runs with realistic natural and anthropogenic radiative forcing from 1900–2005 (Historical), and 3) projections of future climate from 2006–2100 with external radiative forcing being prescribed by four different Representative Concentration Pathways (RCP 2.6, 4.5, 6.0, and 8.5). Monthly anomalies are calculated relative to individual model’s centennial climatology for each experiment and smoothed using a five-month running mean.

### Definition of the CP and EP El Niño

For the observations and model simulations, when both the five-month running mean Niño3 (5°S-5°N, 150°W-90°W) and Niño4 (5°S-5°N, 160°E-150°W) SST anomalies exceed 0.5 °C, the EP El Niño is defined if the Niño3 SST anomaly is greater than the Niño4 anomaly. When the Niño4 SST anomaly is larger, it is defined as a CP El Niño^2^,^5^. Because of the CMIP5 models’ poor performance in simulating the seasonal phase-locking of El Niño amplitude^46^, we do not use the observed peak month to represent the peak phase of modelled El Niño. Instead, we choose the peak month for individual model of each experiment separately. The peak month of either the observed or modelled El Niño is determined when the standard deviation of Niño3.4 (5°S-5°N, 170°W-120°W) SST anomaly reaches a maximum across the 12 calendar months. For simplicity, we use the five-month running mean Niño3 and Niño4 SST anomalies centered in the peak month to classify the EP and CP El Niño. During the period of 1950-2013 in the observations (December is the peak month), seven EP events (1951, 1965, 1972, 1976, 1982, 1991, 1997) and 16 CP events (1953, 1958, 1963, 1968, 1969, 1977, 1979, 1986, 1987, 1990, 1994, 2002, 2003, 2004, 2006, 2009) are classified. These are similar to previous results using different classification methods based on December-February mean SST anomalies^47^.

### Power spectral analysis and band-pass filtering

All the analyses are conducted using the NCAR Command Language software package (https://www.ncl.ucar.edu, where details for each method are available). Power spectral analysis is performed based on the 5-month running mean Niño3, Niño4, CP and EP El Niño indices. The statistical confidence interval of the power spectra is estimated with a theoretical Markov red-noise spectrum using the lag-1 autocorrelation. The power spectra of both the Niño4 and all CP El Niño indices display a decadal peak that is marginally significant at 10% significance level (Fig. 1b), probably owing to the short record of observations. To decompose the interannual and decadal variability, we apply the Lanczos filter^48^ to the five-month running mean SST anomaly. We generate the low-pass filtered SST anomaly with a cut-off period of seven years using the sharpest response function provided by the NCAR software package. The difference between the original five-month running mean anomaly and the low-pass filtered anomaly gives the band-pass filtered SST anomaly (i.e., five months to seven years). Results based on a cut-off period of eight years or independent high-pass filtering or other popular filtering methods are similar. Classification of the CP and EP El Niño on interannual timescales is performed using the same two criteria described above but based on the band-pass filtered SST anomalies. To assess the impact of secular trends on the frequency of the CP El Niño, the El Niño classification is performed based on detrended and non-detrended SST anomalies, respectively. Note that, in the RCP8.5 scenario projection, the centennial trend of the SST warming is so strong that most of CP and EP El Niños are counted in the second half of the 21^st^ century in the non-detrended case.

## Additional Information

**How to cite this article**: Sullivan, A. *et al*. Robust contribution of decadal anomalies to the frequency of central-Pacific El Niño. *Sci. Rep.* **6**, 38540; doi: 10.1038/srep38540 (2016).

**Publisher's note:** Springer Nature remains neutral with regard to jurisdictional claims in published maps and institutional affiliations.

## Supplementary Material

Supplementary Information
