# Supplementary material for: Robust contribution of decadal anomalies to the frequency of central-Pacific El Niño
Source: Sci Rep. 2016 Dec 5;6:38540. doi: 10.1038/srep38540 (PMC5137076; doi:10.1038/srep38540)
Supplement: Supplementary Information [file srep38540-s1.pdf]

Supplementary information:

Robust contribution of decadal anomalies to the frequency of central-Pacific El Niño

Arnold Sullivan[1], Jing-Jia Luo[2*], Anthony C. Hirst[2], Daohua Bi[1], Wenju Cai[1] & Jinhai He[3]

[1]CSIRO Marine and Atmospheric Research, Aspendale, Victoria, Australia

[2]Bureau of Meteorology, Victoria, Australia

[3]Nanjing University of Information Science and Technology, Nanjing, China

[*]Corresponding author. E-mail: j.luo@bom.gov.au

This supplementary information file includes:

Supplementary Tables 1-4
Supplementary Figures 1-8

Supplementary Tables

Supplementary Table 1: Cross correlation coefficients among popularly used EP and CP El Niño indices. a) Correlations between the EP/CP El Niño indices (which are named as in Fig. 1) and Niño3/ Niño4 indices. **b-c**) Correlations among the EP and CP El Niño indices. Results are based on the detrended 5-month running mean SST anomalies during 1950-2013. Correlations with a magnitude above 0.35 are significant at the 5% level based on 30 degrees of freedom estimated using lag-correlation. Correlations are similar for non-detrended SST anomalies.

Supplementary Table 1a

	Niño3.4	Niño3	Niño4	EP_{new}	EP_{tEOF}	EP_{REOF}	EP_{EOF}	EP_{pEOF}	EP_{prEOF}
Niño3.4		0.94	0.91	0.69	0.59	0.96	0.96	0.36	0.74
Niño3			0.76	0.88	0.81	0.95	0.97	0.63	0.91
Niño4				0.37	0.28	0.87	0.86	0.0	0.49
CP_{new}	0.63	0.36	0.88	-0.11					
CP_{tEOF}	0.77	0.56	0.93		0.0				
CP_{REOF}	0.06	-0.23	0.38			0.0			
CP_{EOF}	0.13	-0.17	0.46				0.0		
CP_{pEOF}	0.69	0.45	0.85					-0.35	
CP_{prEOF}	0.29	0	0.61						-0.27
EP_{new} + CP_{new}	0.99								

Supplementary Table 1b

	EP_{tEOF}	EP_{REOF}	EP_{EOF}	EP_{pEOF}	EP_{prEOF}
EP_{new}	0.95	0.74	0.77	0.90	0.96
EP_{tEOF}		0.68	0.71	0.95	0.95
EP_{REOF}			1.0	0.48	0.84
EP_{EOF}				0.51	0.86
EP_{pEOF}					0.86

Supplementary Table 1c

	CP_{new}	CP_{tEOF}	CP_{REOF}	CP_{EOF}	CP_{pEOF}
CP_{tEOF}	0.92				
CP_{REOF}	0.71	0.65			
CP_{EOF}	0.78	0.71	0.98		
CP_{pEOF}	0.89	0.96	0.71	0.78	
CP_{prEOF}	0.93	0.83	0.94	0.96	0.84

Supplementary Table 2: Ratio of decadal variance to interannual variance in El Niño indices. The ratios are calculated between the decadal (greater than seven years) variance and the interannual (five months to seven years) variance for the Niño3, Niño4, EP, and CP El Niño indices, respectively. The different EP and CP El Niño indices are named as in Fig. 1. Results are calculated with detrended anomalies during 1950-2013.

Supplementary Table 2a

	Niño3	EP_{new}	EP_{tEOF}	EP_{REOF}	EP_{EOF}	EP_{pEOF}	EP_{prEOF}
ratio	0.42	0.51	0.70	0.49	0.45	1.21	0.52

Supplementary Table 2b

	Niño4	CP_{new}	CP_{tEOF}	CP_{REOF}	CP_{EOF}	CP_{pEOF}	CP_{prEOF}
ratio	0.96	1.86	1.69	1.74	1.72	1.94	1.77

Supplementary Table 3: CMIP5 models used in analysis. These models' data were available at the time of analysis. The first member of each model for each experiment is analyzed. Detailed information on the model and experiment is available at http://pcmdi9.llnl.gov/.

Model name	piControl	Historical	RCP2.6	RCP4.5	RCP6.0	RCP8.5
ACCESS1-0	X	X		X		X
ACCESS1-3	X	X		X		X
bcc-csm1-1	X	X	X	X	X	X
CanESM2	X	X	X	X		X
CNRM-CM5	X	X	X	X		X
CSIRO-Mk3-6-0	X	X	X	X	X	X
GFDL-ESM2G	X	X	X	X		X
GFDL-ESM2M	X	X	X	X	X	
GISS-E2-H	X	X	X			
GISS-E2-R	X	X				
inmcm4	X	X		X		X
IPSL-CM5A-LR	X	X		X	X	X
IPSL-CM5A-MR		X		X		X
MIROC5	X	X	X	X	X	X
MIROC-ESM	X	X	X	X	X	X
MIROC-ESM-CHEM		X	X	X	X	X
MPI-ESM-LR	X	X	X	X		X
MPI-ESM-MR		X				
MRI-CGCM3	X	X	X	X	X	X
NorESM1-M	X	X	X	X	X	X
Total	17	20	13	17	9	16

Supplementary Table 4: Observed and simulated ratio of the decadal variance to the total variance of the Niño3 and Niño4 index. The ratio of CMIP5 Historical, piControl, and four RCP scenario simulations denotes the average value of all model runs for each simulation. Results are calculated with detrended anomalies.

	observed	piControl	Historical	RCP2.6	RCP4.5	RCP6.0	RCP8.5
Niño4	0.42	0.37	0.41	0.42	0.40	0.45	0.42
Niño3	0.26	0.23	0.25	0.27	0.24	0.27	0.24

Supplementary Figures

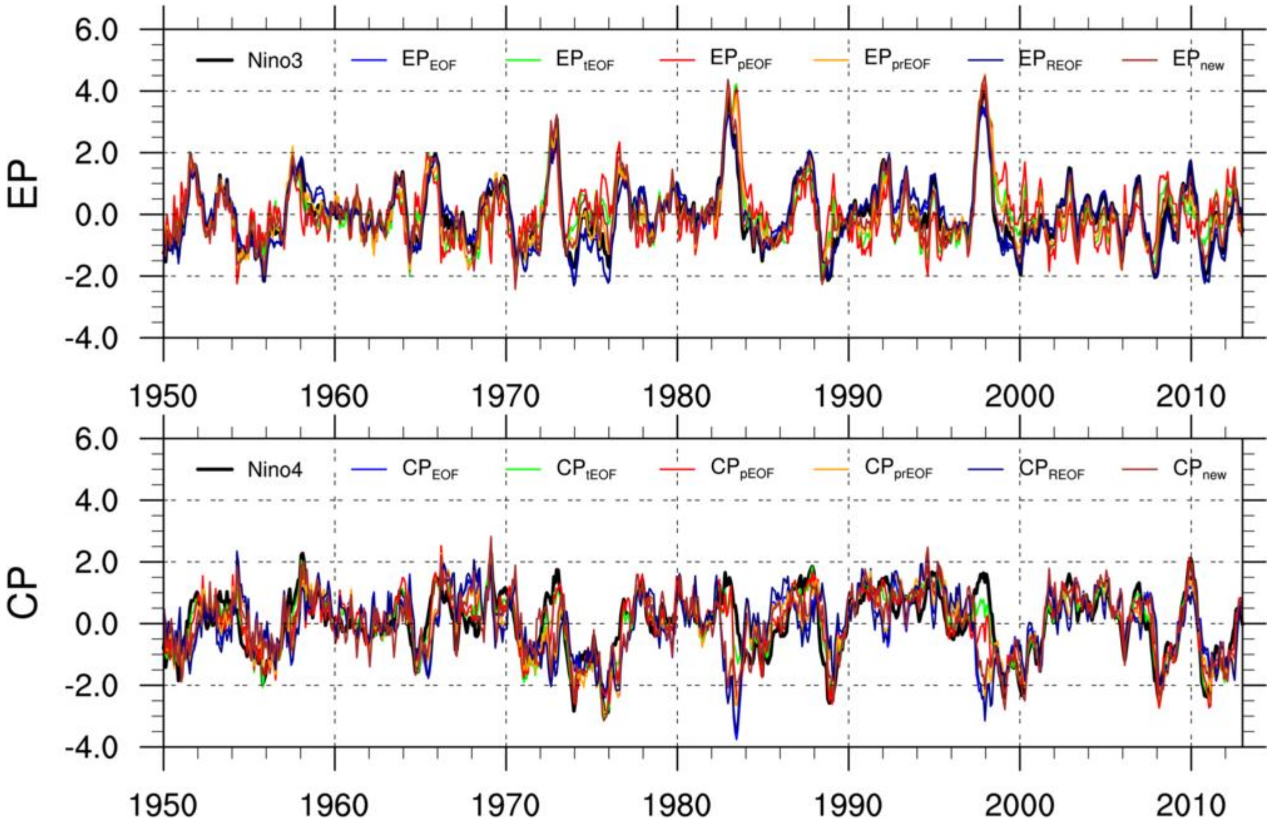

Supplementary Fig. 1: **Normalized Nino3, Nino4, EP and CP El Niño indices.** Results are based on the detrended 5-month running mean SST anomalies during 1950-2013. The different EP and CP El Niño indices are named as in Fig. 1. This figure is created using NCAR Command Language software package version 6.3.0 (https://www.ncl.ucar.edu).

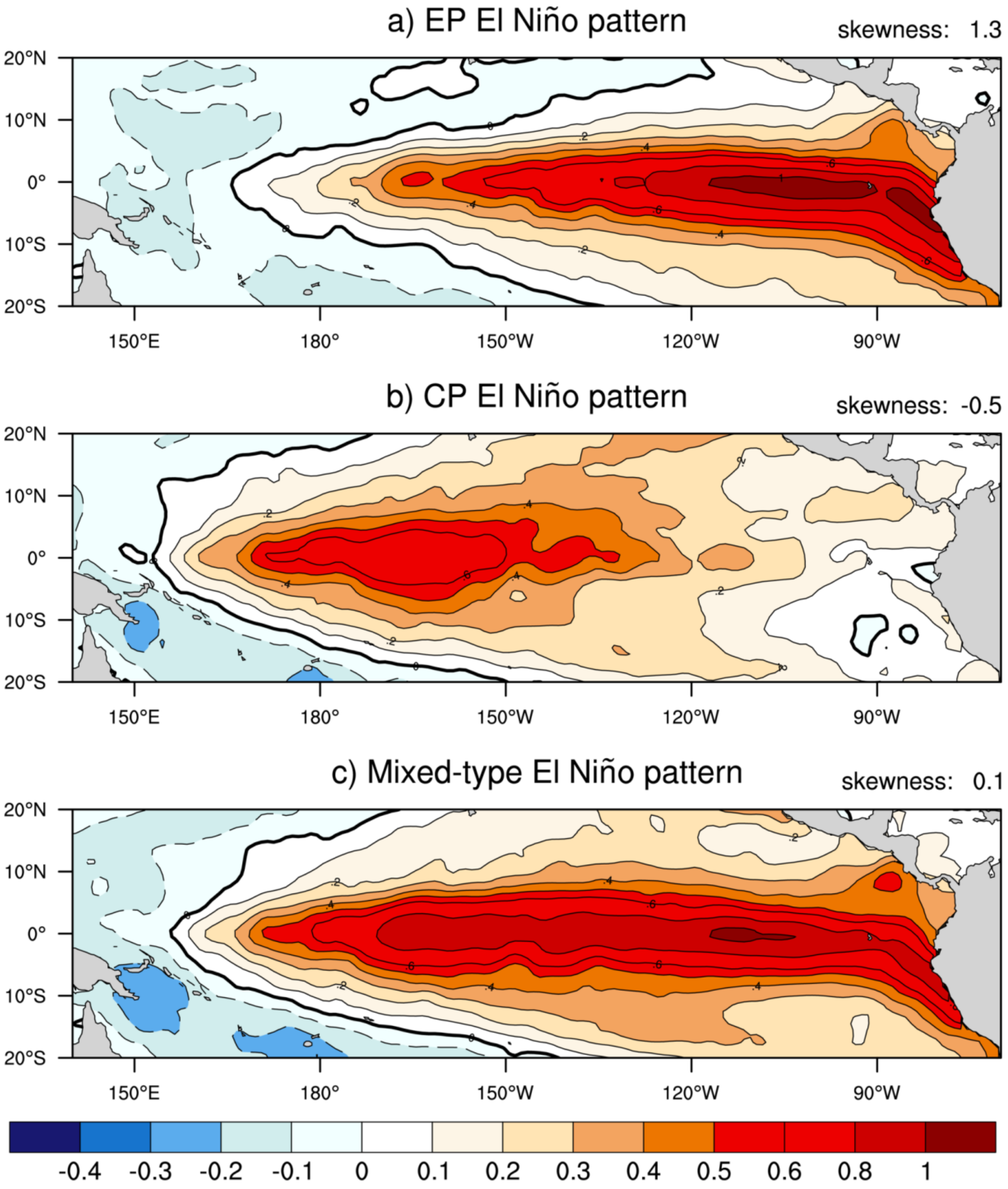

Supplementary Fig. 2: **SST anomaly patterns associated with the EP_{new}, CP_{new} and mixed type of El Niño during 1950-2013.** The SST anomalies are linearly regressed onto the standardized and detrended EP_{new}, CP_{new}, and (EP_{new} + CP_{new}) indices (see the text for definitions). The skewness of each index is indicated above each panel. This figure is created using NCAR Command Language software package version 6.3.0 (https://www.ncl.ucar.edu).

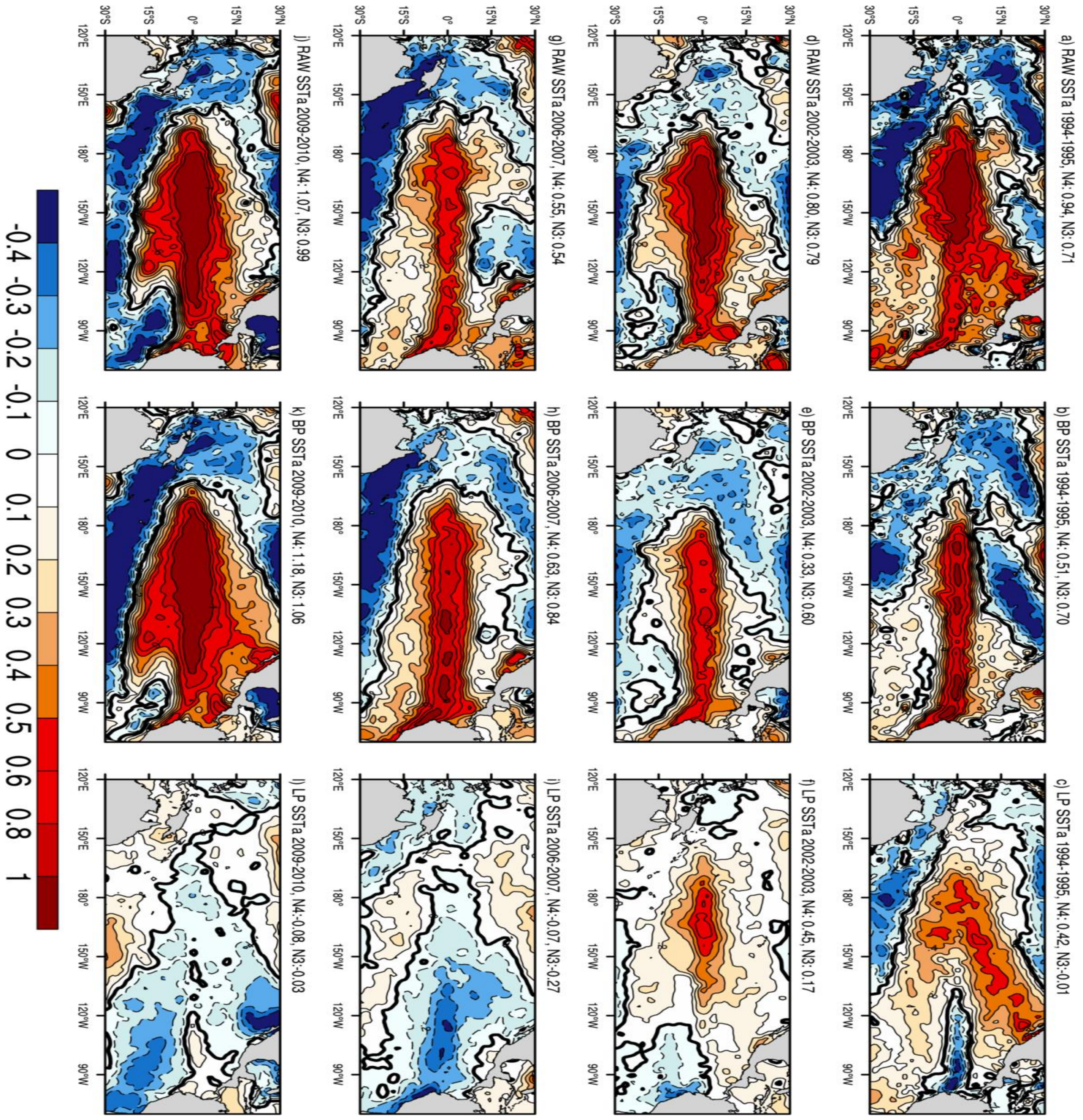

Supplementary Fig. 3: Decomposed SST anomalies of four CP El Niño events at interannual and decadal timescales. (**a-c**) Spatial patterns (October 1994 to February 1995) based on monthly raw SST anomalies, band-pass (BP, five months to seven years) and low-pass (LP, greater than seven years) filtered SST anomalies. (**d-f**), (**g-i**) and (**j-i**) As in (**a-c**), but for the CP El Niño events in 2002/03, 2006/07, and 2009/10. The Niño3 and Niño4 SST anomalies (˚C) decomposed at different timescales are indicated in panel titles. This figure is created using NCAR Command Language software package version 6.3.0 (https://www.ncl.ucar.edu).

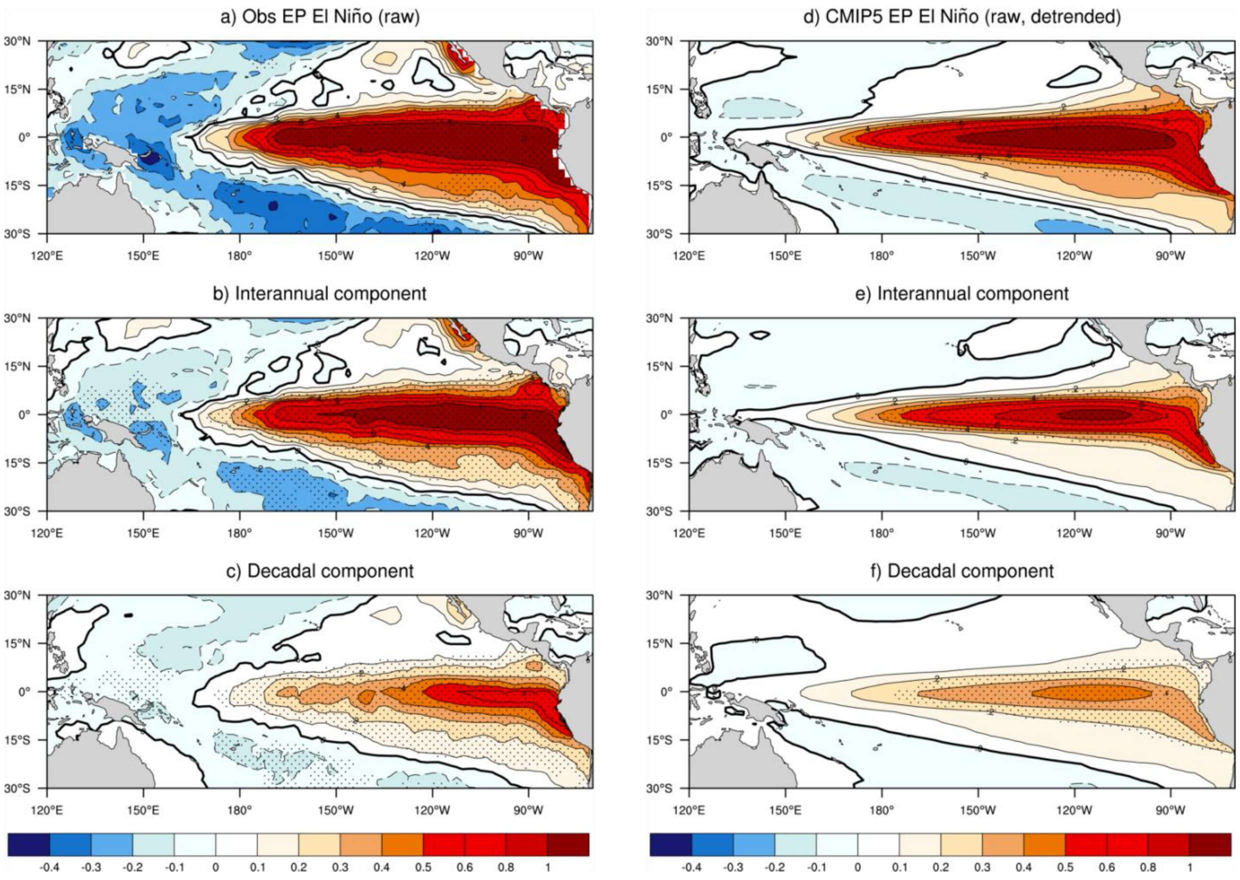

Supplementary Fig. 4: Composite maps of EP El Niños at different timescales. (**a-c**) Observed SST composites from detrended HadISST (1950-2013). Stippling indicates the 5% significance level according to a Student's t-test. Results based on non-detrended data are similar. (**d-f**) As in (**a-c**), but for the CMIP5 model simulations (Methods). This figure is created using NCAR Command Language software package version 6.3.0 (https://www.ncl.ucar.edu).

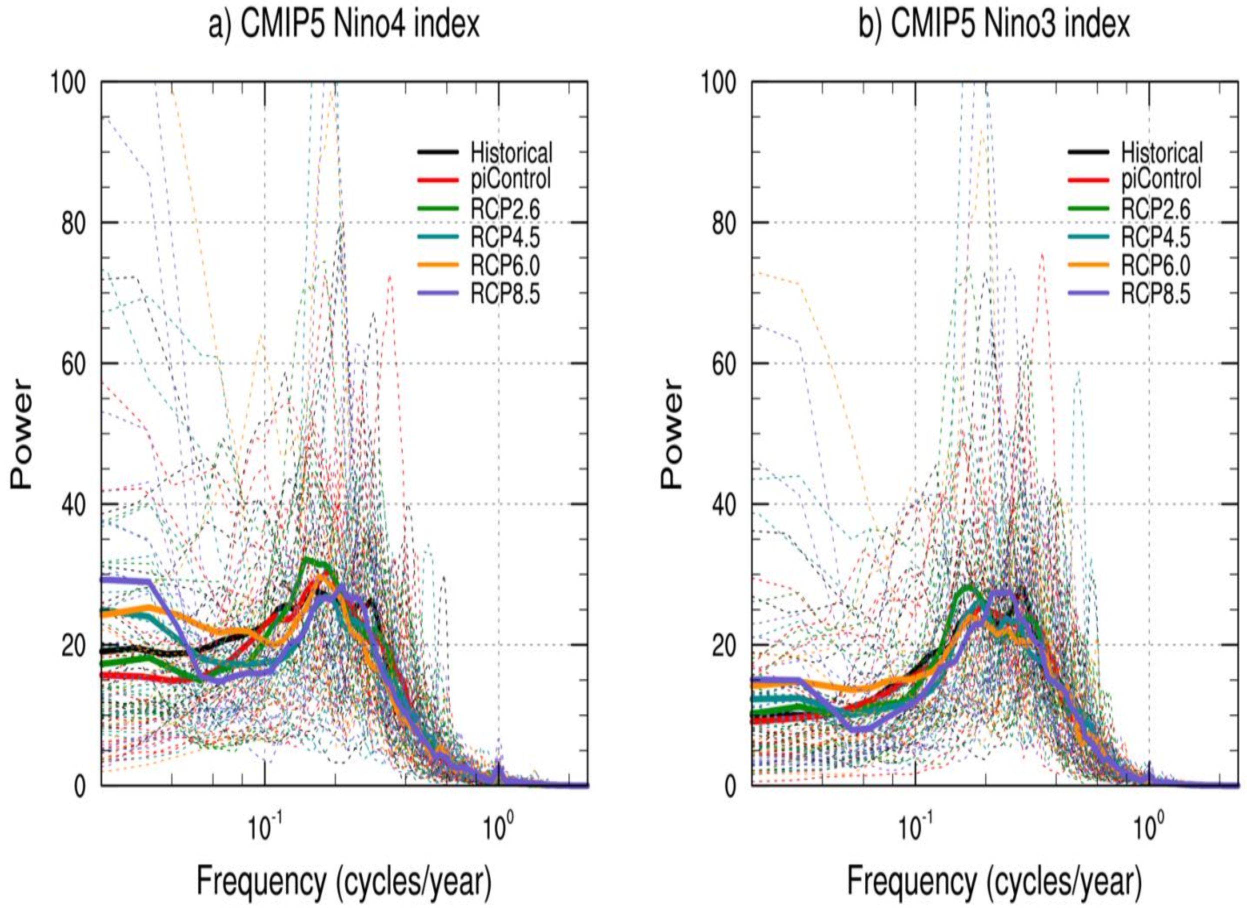

Supplementary Fig. 5: Power spectra of normalized Niño4 and Niño3 indices based on centennial simulations of CMIP5 Historical, piControl, and four RCP scenarios. Dashed thin lines denote the results of individual model runs and solid thick lines indicate the averaged power spectra of the model runs for each simulation.

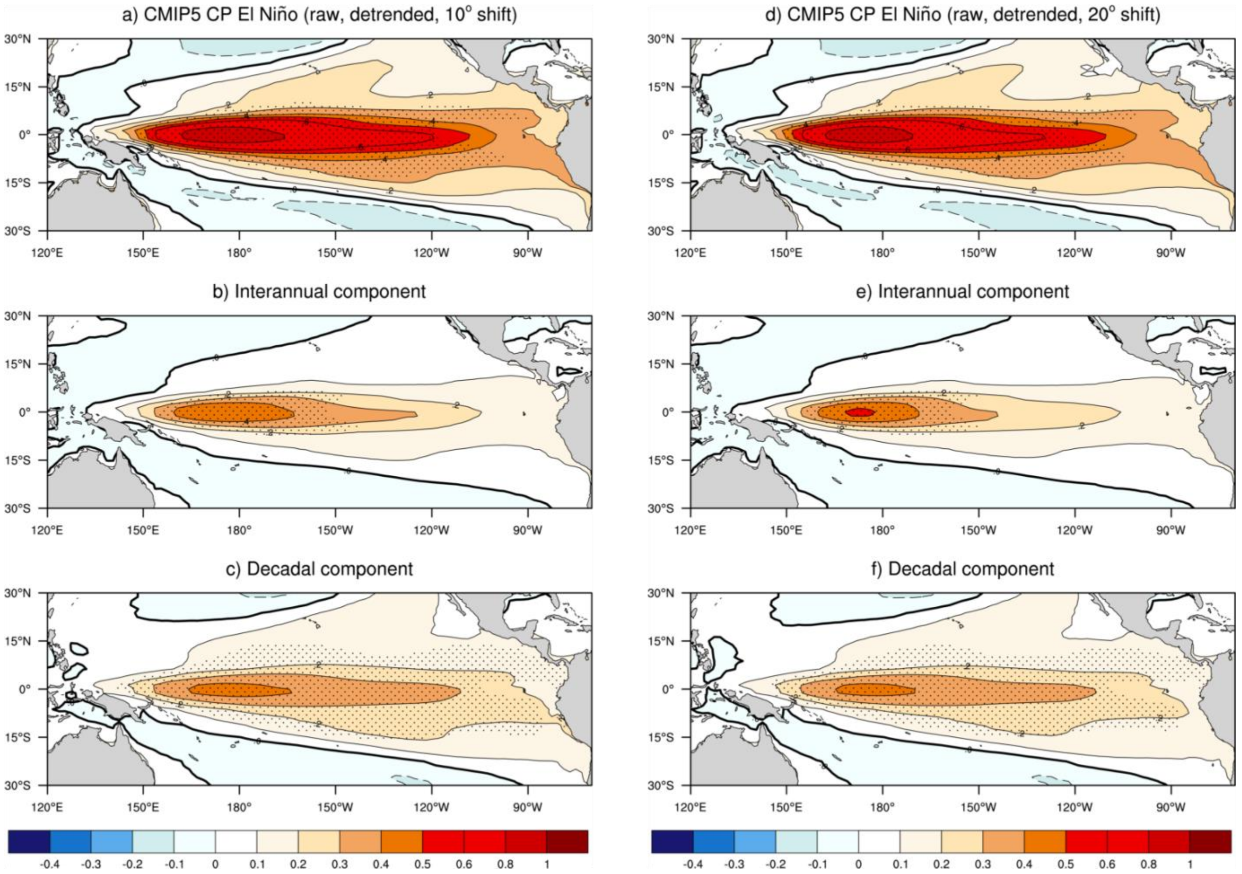

Supplementary Fig. 6: Composite maps of CP El Niños at different timescales based on CMIP5 model simulations. As in Figs. 3a-3c, but with the models' Niño3 and Niño4 indices shifted westward for (**a-c**) 10˚ and (**d-f**) 20˚ of longitude. Stippling indicates the 5% significance level according to a Student's t-test. This figure is created using NCAR Command Language software package version 6.3.0 (https://www.ncl.ucar.edu).

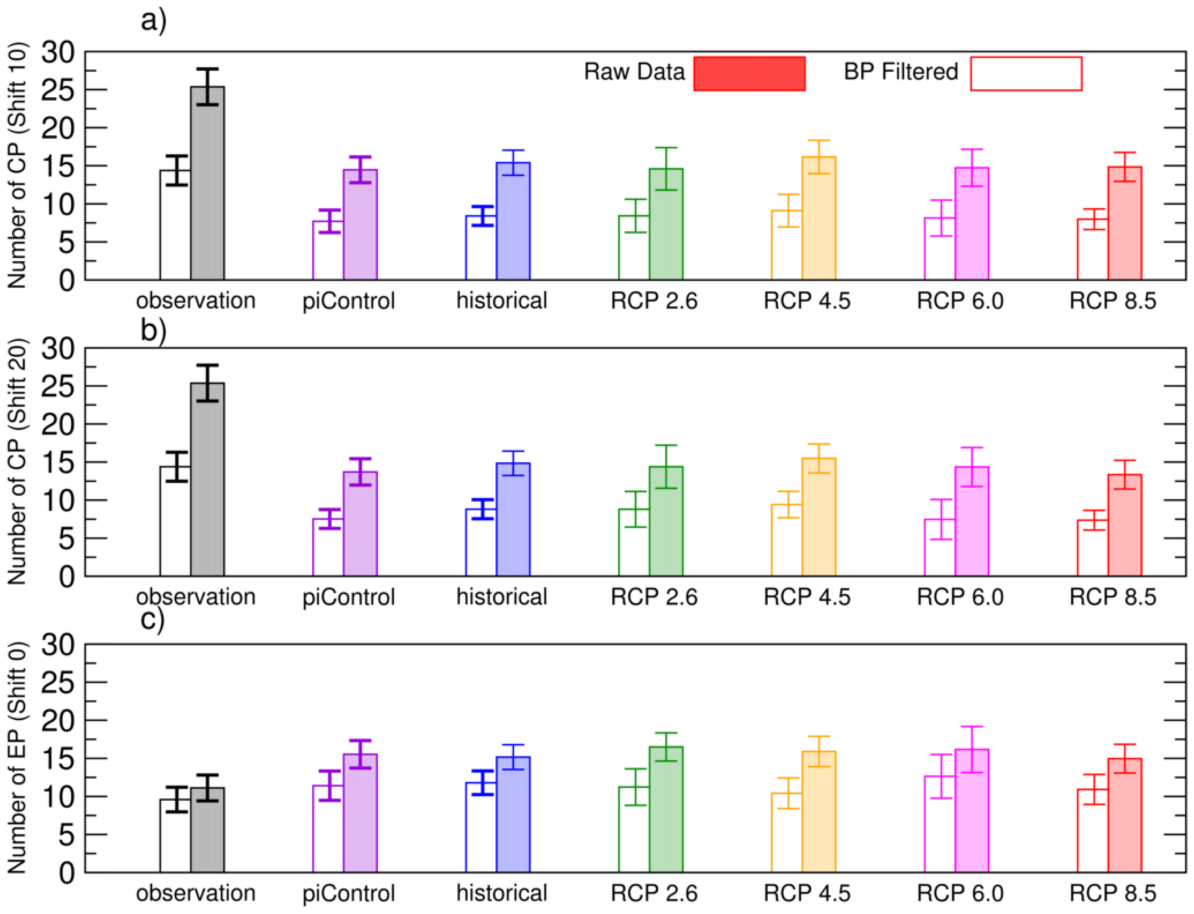

Supplementary Fig. 7: **Impact of decadal anomalies on the frequency of the CP and EP El Niño.** As in Fig. 4a, but the frequency of the CP El Niño per 100 years is based on the models' Niño3 and Niño4 indices being shifted westward for (**a**) 10˚ and (**b**) 20˚ of longitude. Error bars indicate the 95% confidence intervals. (**c**) As in Fig. 4a, but for the frequency of the EP El Niño per 100 years. This figure is created using Grace Version 5.1.23 (http://plasma-gate.weizmann.ac.il/Grace/).

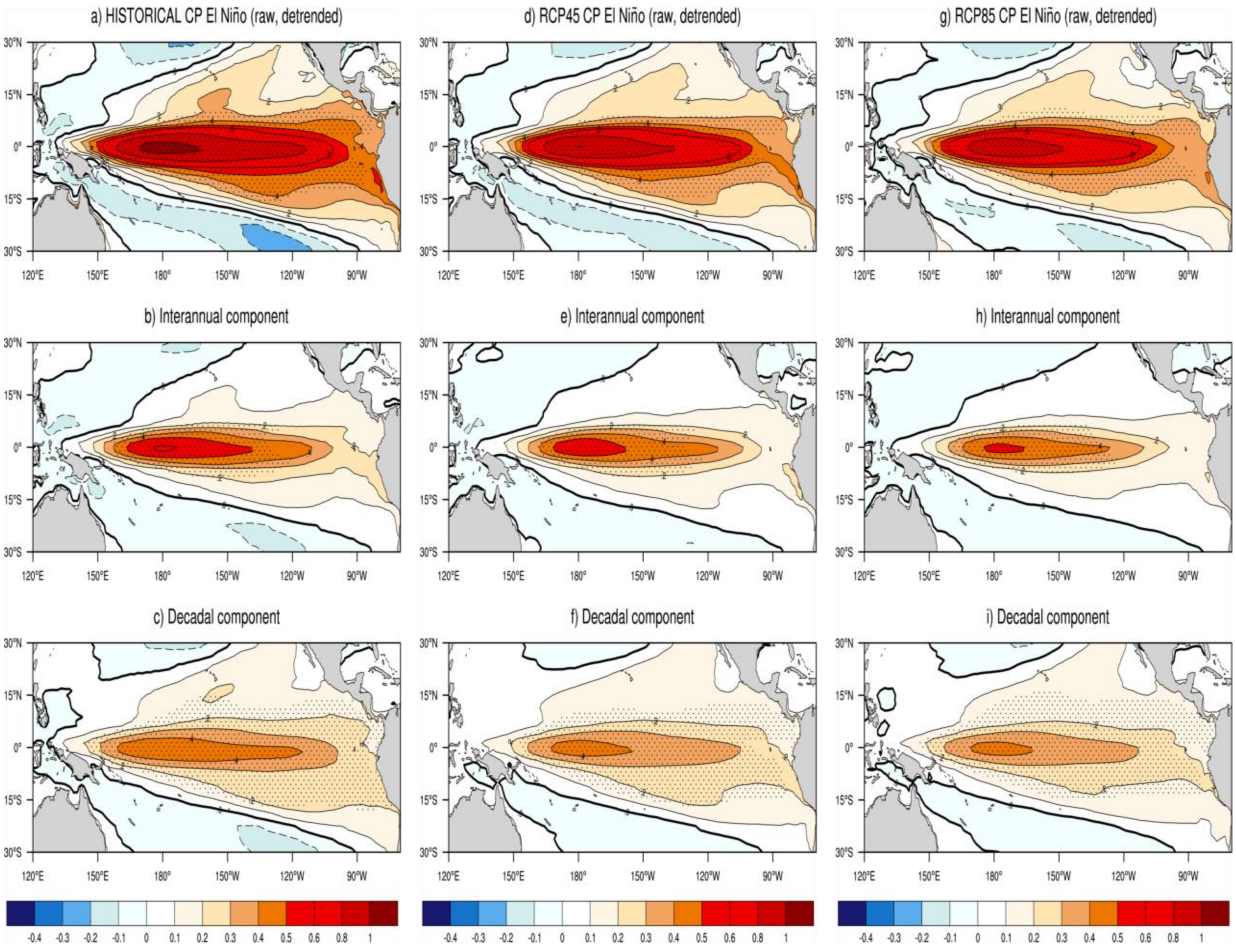

Supplementary Fig. 8: Composite maps of CP El Niños at different timescales based on individual CMIP5 model simulations. As in Figs. 3a-c, but for the results based on (**a-c**) Historical, (**d-f**) RCP4.5 and (**g-i**) RCP8.5 experiments, respectively. This figure is created using NCAR Command Language software package version 6.3.0 (https://www.ncl.ucar.edu).
